# *Borrelia garinii* and *Rickettsia monacensis* in *Ixodes ricinus* Ticks, Algeria

**DOI:** 10.3201/eid2010.140265

**Published:** 2014-10

**Authors:** Wassila Benredjem, Hamza Leulmi, Idir Bitam, Didier Raoult, Philippe Parola

**Affiliations:** Université d’El Tarf, El Tarf, Algeria (W. Benredjem);; Aix Marseille Université, Marseille, France (H. Leulmi, D. Raoult, P. Parola);; Université de Boumerdes, Boumerdes, Algeria (I. Bitam)

**Keywords:** Borrelia garinii, Rickettsia monacensis, Ixodes ricinus, ticks, rickettsia, bacteria, Lyme disease, Lyme borreliosis, Algeria

**To the Editor:** Lyme disease (Lyme borreliosis) is caused by a group of related spirochetes (*Borrelia burgdorferi* sensu lato) that include ≥11 species (*1*). In northern Africa, the main vector of Lyme disease in Europe (*Ixodes ricinus* ticks) is also present, and this disease has been suspected to be present in this region of Africa (*2*). Twenty-one cases of Lyme disease were reported in Algiers, Algeria, during 1996–1999 (*3*). However, these cases were diagnosed by detection of only serum antibodies against *B. burgdorferi* by ELISA without confirmation by Western blotting.

*I. ricinus* ticks are also known to harbor spotted fever group rickettsiae, including *Rickettsia monacensis*, which was detected in Algeria in 2009 (*4*). This rickettsia has been recently identified as a human pathogen in Spain and Italy (*5*).

To investigate Lyme disease and tickborne rickettsioses transmitted by *I. ricinus* ticks in northeastern Algeria, we collected ticks by using the flag method in El Ghora (Bougous, El Tarf) (36°39′34″N, 8°22′10″E). Ectoparasites were collected in March 2012 and identified to genus and species by using taxonomic morphologic keys (*6*).

Total genomic DNA was isolated by using the QIAamp Tissue Kit (QIAGEN, Hilden, Germany) and BioRobot EZ1 (QIAGEN) as described by the manufacturer. DNA was used as template for quantitative real-time PCR. We used the RKND03 system, which is specific for the *gltA* gene of *Rickettsia* spp. (*7*), and the Bor16S system, which is specific for the *rrs* gene of *Borrelia* spp. (*8*). Real-time PCRs were performed by using the CFX96 Real Time System C1000 Touch Thermal Cycler (Bio-Rad Laboratories, Singapore).

Positive results were confirmed by using a standard PCR specific for the *ompA* gene of *Rickettsia* spp. and the 16S rRNA and *flaB* genes of *Borrelia* spp. (*8*). We used bacteria-free DNA of *Rhipicephalus sanguineus* ticks reared in our laboratory colonies as a negative control and DNA of *B. crocidurae* and *R. montanensis*, which are not known to be associated with *I. ricinus* ticks, as a positive control.

PCR amplification was verified by electrophoresis of products on 2% agarose gels. Products were purified by using a NucleoFast 96 PCR plate (Macherey-Nagel EURL, Hoerdt, France) as recommended by the manufacturer. Purified PCR products were sequenced by using the same primers as for a standard PCR and the BigDye version 1–1 Cycle Ready Reaction Sequencing Mixture (Applied Biosystems, Foster City, CA, USA) in the ABI 31000 automated sequencer (Applied Biosystems). Sequences were assembled and analyzed by using ChromasPro version 1.34 software (Technelysium Pty. Ltd., Tewantin, Queensland, Australia).

Ninety-four ticks were collected by using the dragging method; these ticks belonged to 2 species: 85.1% (80/174) were *I. ricinus* ricks (43 females, 22 males, and 15 nymphs) and 14.9% (14/94) were *Rh. sanguineus* adult ticks (11 females and 3 males). We screened only the 80 *I. ricinus* ricks. *Rh. sanguineus* ticks were kept alive to establish laboratory colonies for other experiments. Overall, 5.0% (4/80) of *I. ricinus* ticks were positive for *Borrelia* spp. and 8.75% (7/80) were positive for *Rickettsia* spp.

Using a standard PCR specific for the *flaB* gene, we identified *B. garinii* in all ticks positive by quantitative real-time PCR (100% similarity, 736/736 bp) (GenBank accession no. CP003151.1). Using a standard PCR specific for the *ompA* gene of *Rickettsia* spp., we identified *R. monacensis* (100% similarity 760/760 bp) (GenBank accession no. FJ919640.1).

We have detected *B*. *garinii,* a cause of Lyme disease, in Algeria in *I. ricinus* ticks by using a standard PCR and sequencing methods. We also confirmed the presence of *R. monacensis* in this country.

*Borrelia* spp. have been detected in *I. ricinus* ticks in Tunisia and Morocco (*2*,*9*), and *B. lusitaniae* was found to be predominant (97% of *Borrelia* spp. in Tunisia and 93% in Morocco). However, *B. garinii* was also present (*2*,*9*,*10*). In Tunisia, 1/16 *I. ricinus* ticks were positive for *B. garinii* (*2*,*9*). In Morocco, 3 (3.6%) of 82 were positive for *B. burgdorferi* sensu stricto and 3 (3.6%) of 82 were positive for *B. garinii* (*9*). However, in these studies, *Borrelia* spp. were identified by using restriction fragment length polymorphism analysis (*2*,*9*).

*B*. *garinii* is the most neurotropic of the genospecies of *B. burgdorferi* sensu lato; it causes meningopolyneuritis and, rarely, encephalomyelitis (*1*). Clinicians need to be aware of the prevalence of this bacterium in Algeria. Our results help clarify the epidemiology of *B. garinii* in Algeria. *R. monacensis* is an agent of tickborne diseases that was detected in Algeria in 2009 (*4*). The few cases that have been described were characterized by influenza-like symptoms, fever, an inoculation eschar, and a generalized rash (*5*).

In northern Africa, the risk areas for Lyme disease and infection with *R. monacensis* include cool and humid areas in the Atlas Mountains. In this region, humans can come in contact with *I. ricinus* ticks, and these ticks might play a major role in transmission of *B. garinii* and *R. monacensis*.
